# Ischemic Stroke Temporally Associated With New-Onset Atrial Fibrillation: A Population-Based Registry-Linkage Study

**DOI:** 10.1161/STROKEAHA.123.044448

**Published:** 2023-12-08

**Authors:** Jukka Putaala, Konsta Teppo, Olli Halminen, Jari Haukka, Paula Tiili, Jussi Jaakkola, Elin Karlsson, Miika Linna, Pirjo Mustonen, Janne Kinnunen, Tuomas Kiviniemi, Aapo Aro, Juha Hartikainen, Juhani K.E. Airaksinen, Mika Lehto

**Affiliations:** Department of Neurology (J.P., P.T., J.K.), Helsinki University Hospital and University of Helsinki, Finland.; Heart and Lung Center (A.A.), Helsinki University Hospital and University of Helsinki, Finland.; Heart Center, Turku University Hospital and University of Turku, Finland (K.T., J.J., P.M., T.K., K.E.J.A.).; Department of Industrial Engineering and Management, Aalto University, Espoo, Finland (O.H., M. Linna).; Department of Public Health, University of Helsinki, Finland (J. Haukka).; University of Helsinki, Finland (E.K., M. Lehto).; Heart Center, Kuopio University Hospital and University of Eastern Finland, Finland (J. Hartikainen).; Department of Internal Medicine, Jorvi Hospital and Helsinki University Hospital, Espoo, Finland (M. Lehto).

**Keywords:** atrial fibrillation, humans, intracranial embolism, ischemic stroke, retrospective studies

## Abstract

**BACKGROUND:**

Limited data exist on the temporal relationship between new-onset atrial fibrillation (AF) and ischemic stroke and its impact on patients’ clinical characteristics and mortality.

**METHODS:**

A population-based registry-linkage database includes all patients with new-onset AF in Finland from 2007 to 2018. Ischemic stroke temporally associated with AF (ISTAF) was defined as an ischemic stroke occurring within ±30 days from the first AF diagnosis. Clinical factors associated with ISTAF were studied with logistic regression and 90-day survival with Cox proportional hazards analysis.

**RESULTS:**

Among 229 565 patients with new-onset AF (mean age, 72.7 years; 50% female), 204 774 (89.2%) experienced no ischemic stroke, 12 209 (5.3%) had past ischemic stroke >30 days before AF, and 12 582 (5.8%) had ISTAF. The annual proportion of ISTAF among patients with AF decreased from 6.0% to 4.8% from 2007 to 2018. Factors associated positively with ISTAF were higher age, lower education level, and alcohol use disorder, whereas vascular disease, heart failure, chronic kidney disease cancer, and psychiatric disorders were less probable with ISTAF. Compared with patients without ischemic stroke and those with past ischemic stroke, ISTAF was associated with ≈3-fold and 1.5-fold risks of death (adjusted hazard ratios, 2.90 [95% CI, 2.76–3.04] and 1.47 [95% CI, 1.39–1.57], respectively). The 90-day survival probability of patients with ISTAF increased from 0.79 (95% CI, 0.76–0.81) in 2007 to 0.89 (95% CI, 0.87–0.91) in 2018.

**CONCLUSIONS:**

ISTAF depicts the prominent temporal clustering of ischemic strokes surrounding AF diagnosis. Despite having fewer comorbidities, patients with ISTAF had worse, albeit improving, survival than patients with a history of or no ischemic stroke.

**REGISTRATION:**

URL: https://www.clinicaltrials.gov; Unique identifier: NCT04645537. URL: https://www.encepp.eu; Unique identifier: EUPAS29845.

Atrial fibrillation (AF) is the most common sustained arrhythmia and the most frequent high-risk source of cerebral embolism.^1,2^ While the risk of ischemic stroke attributable to AF is well established, the temporal relationship of this association and precise mechanisms are more disputable. For example, in patients with a pacemaker, there is a lack of temporal association with subclinical AF and ischemic stroke.^3^ AF is often diagnosed only after stroke in routine clinical care or studies utilizing implantable cardiac monitors to systematically screen for AF in patients with cryptogenic ischemic stroke.^4,5^ In many strokes with a temporally remote AF diagnosis, there may not be a strong causal correlation between the conditions. However, recent retrospective hospital-based and prospective community cohort–based studies suggested a profound temporal clustering of ischemic strokes closely surrounding the time of clinical AF diagnosis, referring to a common underlying cardiovascular pathophysiology for these events and, thus, a specific clinical entity.^6,7^

Limited data suggest differences in demographic and comorbid characteristics between patients with ischemic stroke with known AF (KAF), AF diagnosed early after stroke, or later (>6 months) after the stroke.^6,8–10^ Furthermore, some studies suggest increased mortality in both patients with KAF and those with AF diagnosed after stroke (AFDAS), with AFDAS being considered by some to have the highest risk of death,^7,10^ but not all studies.^9^ Definitions for KAF and AFDAS are not standardized, and the exact temporal distance between AF and ischemic stroke is not incorporated in the definitions. Large-scale population-based studies on the temporal relationship between AF diagnosis and ischemic stroke, the characteristics of patients with ischemic stroke temporally associated with AF (ISTAF), and the impact of this relationship on prognosis are lacking.

We hypothesized that the characteristics and mortality of patients with ISTAF would differ from patients with stroke-free AF and patients with new-onset AF and ischemic stroke in their remote history. Furthermore, as early diagnosis of AF, initiation of anticoagulant treatment, and acute stroke therapies have improved over time, we hypothesized that these may also have affected the frequency and mortality related to ISTAF. Thus, we assessed the clinical characteristics, 90-day survival, and their time trends in patients with ISTAF in a population-based cohort.

## METHODS

### Study Population

The FinACAF (Finnish AntiCoagulation in Atrial Fibrillation) is a nationwide retrospective cohort study including all adult patients with AF diagnosis in Finland between 2004 and 2018, as described in detail earlier.^11^ All available national health care register sources were used to identify patients with AF, including hospitalizations and outpatient specialist visits from the Care Register for Health Care, primary health care visits from the Register for Primary Health Care visits, and prescriptions from the National Reimbursement Register upheld by the Social Insurance Institute. *International Classification of Diseases, Tenth Revision*, diagnosis code of I48 (AF or flutter) in any of the registries was required for inclusion. Cohort entry occurred on the date of the first recorded AF diagnosis in any of the registries.

The patient selection process is summarized in Figure S1. For the present analysis, we included a cohort of patients with new-onset AF between 2007 and 2018. To specifically follow only patients with a newly diagnosed AF, we excluded patients with a recorded AF diagnosis or warfarin use between 2004 and 2006 to enable a long enough lookback period to exclude a previous diagnosis of AF.^1^ Ischemic strokes were identified from 2004 to 2018. For ischemic strokes to be classified as new-onset events, hospitalization for ischemic stroke (*International Classification of Diseases, Tenth Revision*, code of I63) and without past ischemic stroke (*International Classification of Diseases, Tenth Revision*, codes of I63 and I69.3) had to be documented in any of the registries before the start date of hospitalization. In Finland, patients with stroke are hospitalized for diagnostic workup and treatment, and the reliability of diagnoses based on hospital registries has been documented as high.^12^ The temporal distance between new-onset AF and new-onset ischemic strokes was calculated by subtracting the date of the ischemic stroke from the date of the first AF diagnosis. Subsequently, based on the temporal distance and stroke status, we categorized the cohort into mutually exclusive groups as follows: (1) ISTAF, ischemic stroke 1 to 30 days prior or 0 to 30 days after the new-onset AF diagnosis registration date; (2) past ischemic stroke >30 days prior the new-onset AF; and (3) no ischemic stroke until 30 days after the new-onset AF.

Baseline patient characteristics were compiled using data from the registries from 2004 until cohort entry with codes and sources used to construct variables for comorbidities described in Table S1. We applied a modified version of the CHA_2_DS_2_-VASc^13^ score so that patients with past ischemic stroke >30 days before new-onset AF gained 2 points and patients with ISTAF gained zero points for the stroke/transient ischemic attack score component. Transient ischemic attacks were not considered in our analysis due to uncertainty about their timing as many of the transient ischemic attack events may have been evaluated in outpatient clinics. Demographic factors considered in the analyses included age, sex, and level of education. The patients’ highest level of education was obtained from Statistics Finland and classified according to the International Standard Classification of Education (ISCED)^14^: category 1: ISCED 0 to 2 (preprimary, primary, and lower secondary education); category 2: ISCED 3 (upper secondary or vocational education); and category 3: ISCED 5 to 8 (tertiary-, bachelor’s-, master’s-, or doctoral-level education). ISCED category 4 does not exist in Finland. Data on the first initiation of oral anticoagulation—warfarin or any of the direct oral anticoagulant (DOAC) apixaban, dabigatran, edoxaban, or rivaroxaban—were obtained from the National Reimbursement Register.

### Follow-Up

The FinACAF database contains follow-up data for all patients until death or December 31, 2018, whichever occurred first. For the present analysis, follow-up was terminated at 90 days from the new-onset AF as we focused on mortality associated with ISTAF and 90 days is an established time point to assess stroke outcomes. Information on vital status and dates of death was retrieved from the National Death Register maintained by the Population Register Centre and Statistics Finland.

### Ethical Considerations and Data Availability

The study adheres to the principles of the Declaration of Helsinki, as revised in 2013. The Ethics Committee of the Medical Faculty of Helsinki University approved the study. Relevant permissions were obtained from the Finnish register holders. According to the Finnish legislation, informed consent was waived due to the retrospective registry study design. Due to the sensitive nature of the data collected for this study, data access requests from qualified researchers trained in human subject confidentiality protocols can be sent to the Finnish national register holders through Findata (https://findata.fi/en/). This article follows the Strengthening the Reporting of Observational Studies in Epidemiology guidelines.^15^

### Statistical Analysis

Statistical analyses were performed with IBM SPSS (version 25.0; IBM Corporation, Armonk, NY) and R (R Core Team, 2023). The distribution and normality of continuous data were assessed. Continuous variables with normal distribution were reported as mean±SD. We used the χ^2^ test to compare differences between proportions and the Student *t* test to compare continuous variables with normal distribution: (1) between ISTAF and no ischemic stroke and (2) between ISTAF and past ischemic stroke. Trends of categorical parameters over time were assessed with the χ^2^ test.

To assess factors independently associated with the ISTAF phenotype in the entire cohort (those with no ischemic stroke and past ischemic stroke as a single comparator group), we applied multivariable logistic regression adjusting for cohort entry year, demographics, and comorbidities and reported results as odds ratio with 95% CI. Assumptions for the model were assessed visually, and multicollinearity was tested. We used cohort entry year (2007–2018) and age (<65, 65–74, and ≥75 years) as categorical variables. All covariates were subsequently forced into the model. We performed a similar sensitivity analysis by excluding patients with past ischemic stroke.

Kaplan-Meier curves were plotted to depict 90-day survival with a log-rank test to compare survival between subgroups. A multivariable Cox proportional hazards model was fitted to assess baseline variables associated with 90-day survival and hazard ratios (HRs) with 95% CI reported for each covariate. Covariates forced into the model included cohort entry year, demographics, comorbidities, and ISTAF phenotype. We also performed a secondary Cox proportional analysis with additional adjustment for oral anticoagulation initiation to assess its effect on the model. In the ISTAF subgroup, we also performed a Cox model to assess factors associated with their survival with cohort entry year, demographics, and comorbidities as covariates. The proportionality of hazards assumptions was assessed visually with no violations observed.

## RESULTS

**Figure 1.**
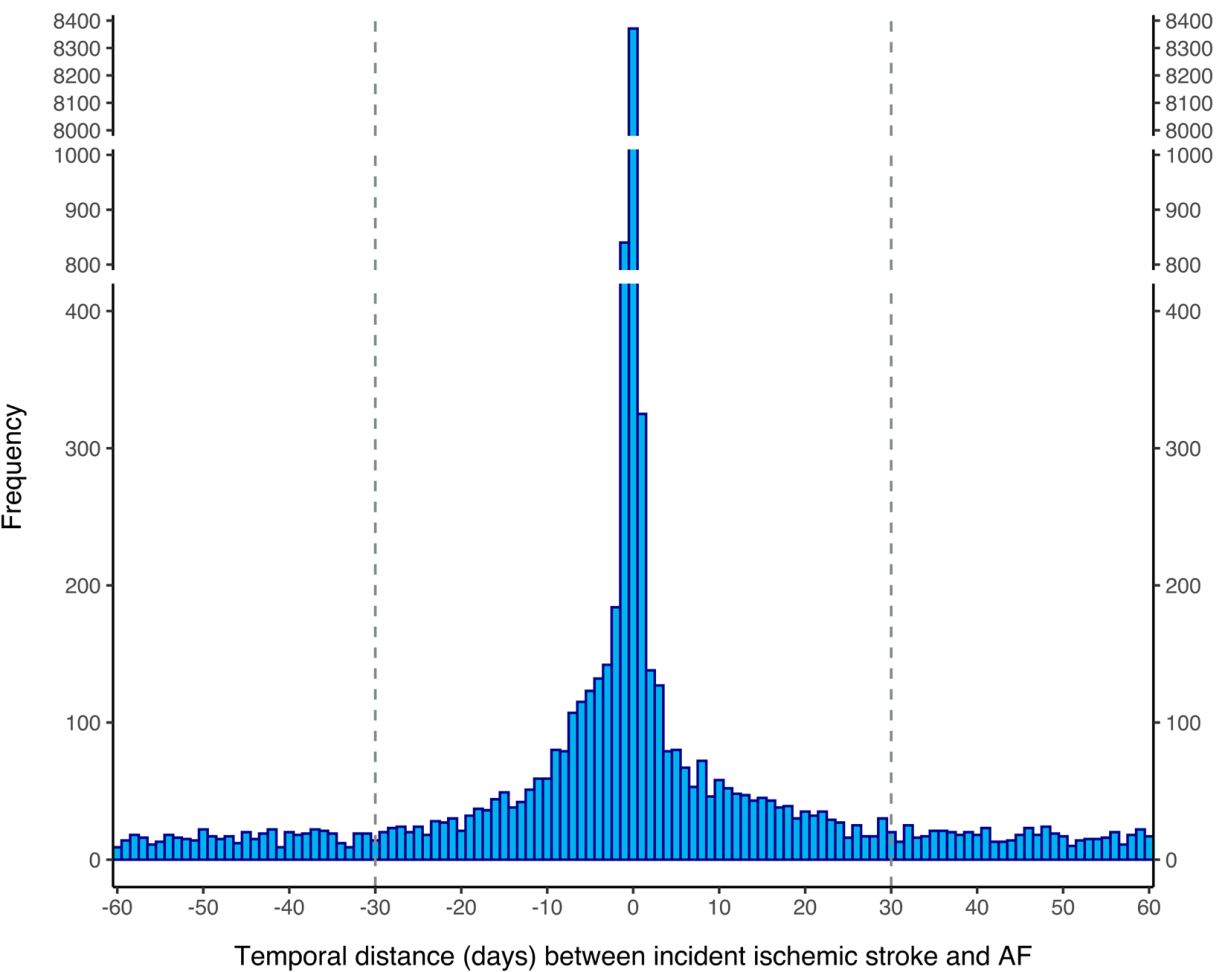
**Frequency of new-onset ischemic strokes according to temporal distance from new-onset atrial fibrillation (AF) within 60 d preceding or following AF (n=25 523).** Dashed lines denote a subcohort selected to represent ischemic strokes temporally associated with AF. *y* axis is gapped at 400 to 800 and 1000 to 8000 to improve the resolution of lower frequencies.

Among the identified 229 565 patients (50.0% female; mean age, 72.7±13.2 years) with new-onset AF between 2007 and 2018, altogether 12 209 (5.3%) patients had past ischemic stroke and 12 582 (5.5%) patients were classified as having ISTAF (Figure S1). Figure 1 displays the distribution of the temporal relationship between new-onset AF and ischemic stroke within the time period from 60 days preceding to 60 days following AF diagnosis, showing flattening of stroke frequencies around and beyond ±30 days from AF, whereas Figure S2 depicts the distance from ischemic strokes and AF in months in the entire cohort. Among patients with ISTAF, 2498 (19.9%) strokes were recorded in 1 to 30 days before AF and 10 084 (80.1%) in 0 to 30 days after AF. Of all patients with new-onset AF, the proportion of ISTAF decreased from 6.0% to 4.8% between 2007 and 2018 (Figure S3).

Compared with patients with AF with no ischemic stroke, patients with ISTAF were older, more often female, and less educated and had more often dyslipidemia, hypertension, prior bleeding, and dementia but had less often history of heart failure, renal failure, liver dysfunction, alcohol abuse, cancer, psychiatric disorder, and DOAC initiations (Table 1). Compared with those with past ischemic stroke, patients with ISTAF were younger and more often female and had higher levels of education and lower prevalence of vascular disease, diabetes, dyslipidemia, heart failure, hypertension, prior bleeding, liver dysfunction, chronic kidney disease, alcohol use disorder, cancer, dementia, psychiatric disorders, and DOAC initiations (Table 1).

**Table 1.**
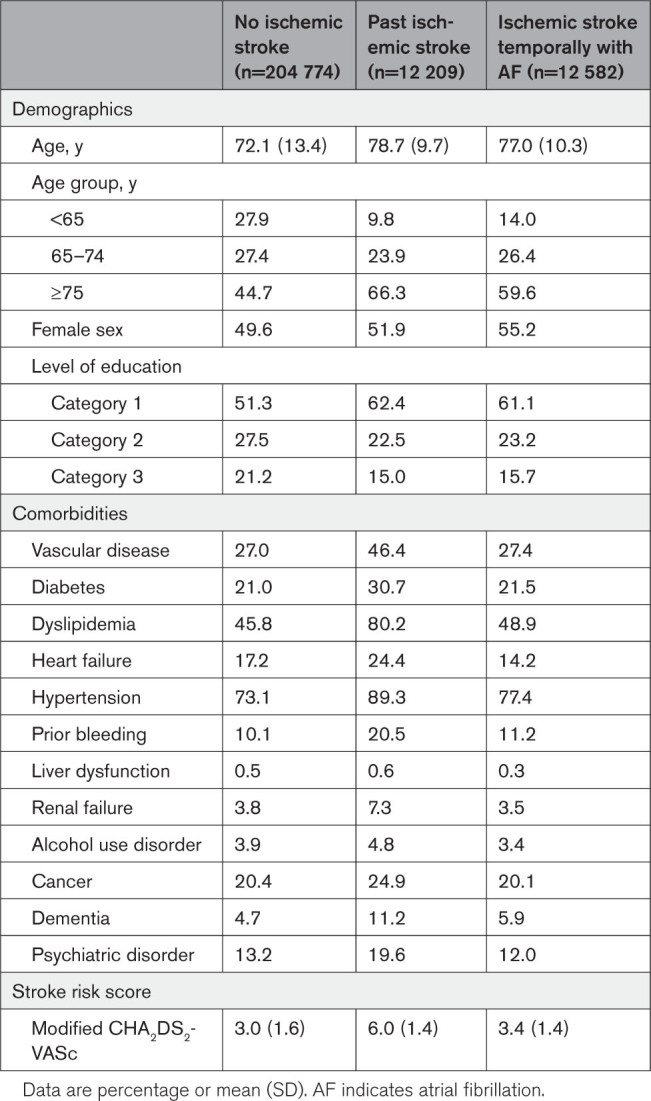
Characteristics of Patients With New-Onset AF Stratified According to Phenotype Based on Temporal Relationship Between Ischemic Stroke and AF

Among patients with ISTAF, 3 of 5 were aged ≥75 years (Table 1). Of the patients with AF aged <65, 65 to 74, and ≥75 years, ISTAF encompassed 2.9%, 5.3%, and 7.0%, respectively. The annual mean age of patients with ISTAF remained unchanged from 2007 to 2018, while the mean age of patients with no ischemic stroke increased steadily and that of patients with past ischemic stroke increased more modestly (Figure S4).

Compared with patients with no ischemic stroke, the mean CHA_2_DS_2_-VASc score was higher in patients with ISTAF (3.4±1.4 versus 3.0±1.6; *P*<0.001), whereas those with past ischemic stroke had a higher CHA_2_DS_2_-VASc score than patients with ISTAF (6.0±1.4; *P*<0.001). The proportion of patients with ISTAF among all patients with AF increased together with the CHA_2_DS_2_-VASc score, and 9 of 10 patients with ISTAF scored ≥2. As ISTAF was not considered in CHA_2_DS_2_-VASc scoring, the highest scores were absent in this group (Figure S5; Table S2). We observed a slight increase in the mean CHA_2_DS_2_-VASc score of patients with ISTAF from 2007 to 2018 (Figure S6).

In the study population, DOAC initiations increased slowly until 2014, with a steep increase thereafter. The proportion of patients with DOAC initiations increased significantly and comparably in those with no ischemic stroke, past ischemic stroke, and ISTAF (Figure S7).

In the logistic regression analysis, including all patients, old age, low level of education, absence of vascular disease, heart failure, renal failure, cancer, and psychiatric disease, and the presence of alcohol use disorder were independently associated with ISTAF (Table 2). In the sensitivity analysis excluding patients with a past ischemic stroke, factors associated with ISTAF were old age, low level of education, the presence of dyslipidemia, hypertension, prior bleeding, and alcohol use disorder, and the absence of vascular disease, heart failure, and cancer (Table S3).

**Table 2.**
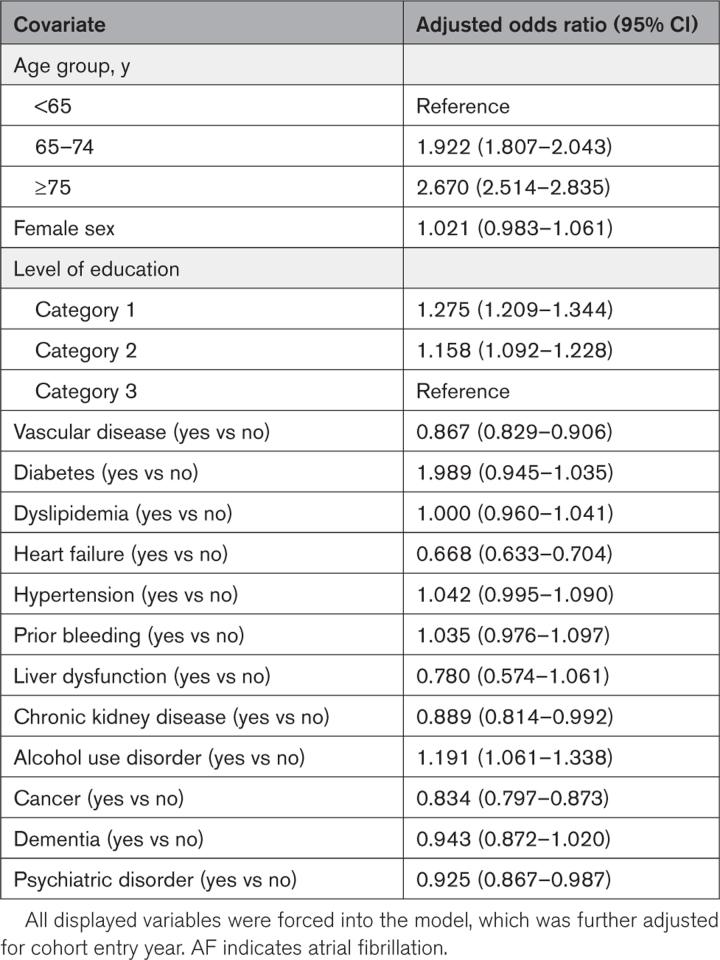
Logistic Regression Analysis on Variables Associated With New-Onset Ischemic Stroke Temporally With New-Onset AF Among All Patients With AF (Those With No Ischemic Stroke and Past Ischemic Stroke as a Single Comparator Group)

**Figure 2.**
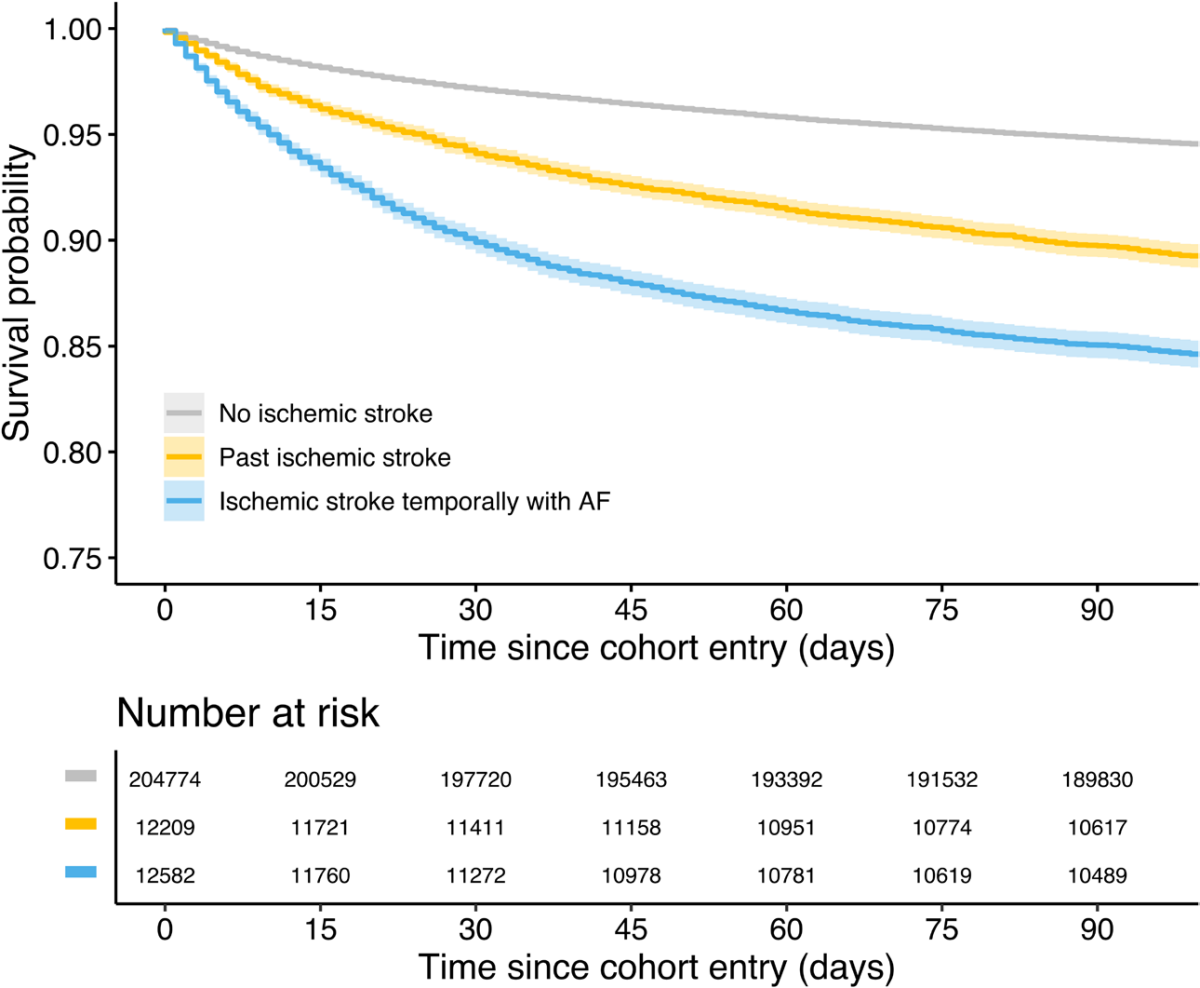
**Ninety-day survival after new-onset atrial fibrillation (AF) stratified by phenotype based on the temporal relationship between ischemic stroke and AF (log-rank *P*<0.001).** Shaded areas denote 95% CIs.

**Figure 3.**
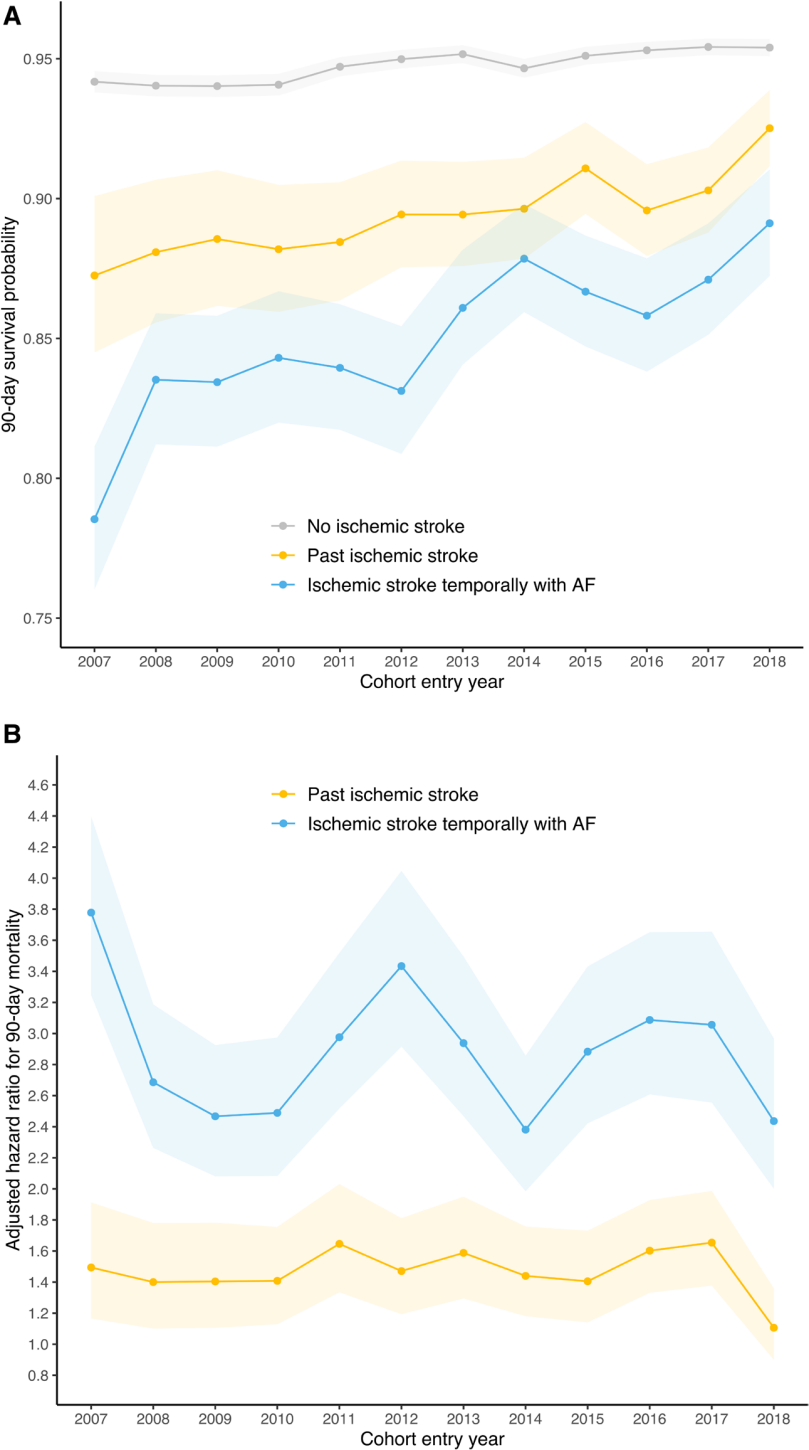
**Time trends of survival and mortality hazard ratios. A**, Time trends of 90-d survival of patients with new-onset atrial fibrillation (AF) in subgroups stratified by phenotype based on the temporal relationship between ischemic stroke and AF. **B**, Time trends of the adjusted hazard ratio for 90-d mortality of patients with ischemic stroke temporally with AF and past ischemic stroke (reference group: no ischemic stroke). Shaded areas denote 95% CIs.

A total of 13 670 patients had died within 90 days from the new-onset AF. The 90-day survival probability was lowest in patients with ISTAF (0.851 [95% CI, 0.844–0.857]) compared with those with past ischemic stroke (0.897 [95% CI, 0.892–0.903]), and patients with no ischemic stroke (0.948 [95% CI, 0.947–0.949]), respectively (Figure 2). We observed an improvement in 90-day survival in all patients, particularly in patients with ISTAF, whose survival probability increased from 0.785 (0.760–0.811) in 2007 to 0.891 (0.872–0.910) in 2018 (Figure 3A). Notably, 11.4% and 1.8% of patients with no ischemic stroke, 9.1% and 2.3% of patients with past ischemic stroke, and 4.8% and 0.4% of patients with ISTAF who died within 90 days had initiated warfarin and DOACs, respectively.

In the adjusted Cox regression analysis, ISTAF was independently associated with 90-day mortality with a ≈3-fold adjusted HR (2.897 [95% CI, 2.757–3.044]) compared with patients with no ischemic stroke and a 1.5-fold HR compared with patients with past ischemic stroke (Table 3). After additional adjustment for the initiation of oral anticoagulation, ISTAF and past ischemic stroke remained associated higher 90-day mortality (HR, 2.641 [95% CI, 2.513–2.775] and 1.268 [95% CI, 1.194–1.347], respectively) Between 2007 and 2018, annual adjusted HRs varied substantially for ISTAF, but the difference between patients with past ischemic stroke and no ischemic stroke remained significant in each year of observation (Figure 3B).

**Table 3.**
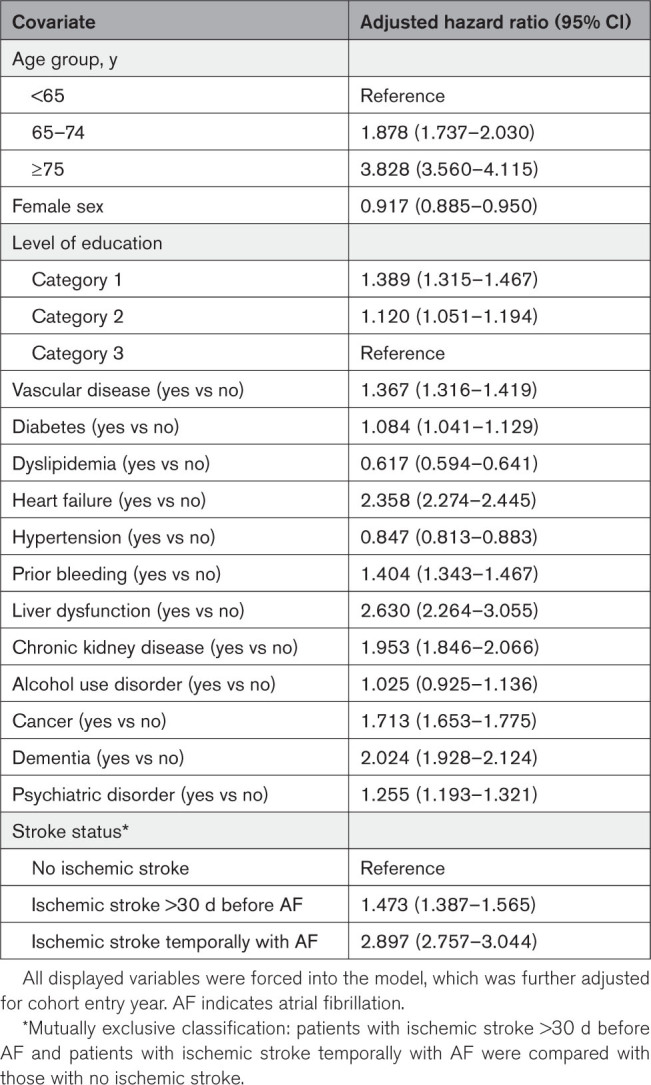
Cox Regression Analysis on the Association of New-Onset Ischemic Stroke Temporally Associated With AF With 90-Day All-Cause Mortality

In patients with ISTAF, the 90-day mortality increased gradually with increasing CHA_2_DS_2_-VASc score (Figure S8). Among demographic and comorbidity variables, mortality was associated with high age, low level of education, vascular disease, diabetes, heart failure, prior bleeding, liver dysfunction, chronic kidney disease, cancer, dementia, and the absence of dyslipidemia (Table S4). In the analysis adjusted for factors not included in the CHA_2_DS_2_-VASc score, 90-day mortality of patients with ISTAF increased with an HR of 1.459 (95% CI, 1.407–1.513) per CHA_2_DS_2_-VASc point increment.

## DISCUSSION

The main findings of this population-based study were (1) new-onset ischemic strokes cluster profoundly with new-onset AF within a ±30-day time window surrounding the AF diagnosis date, forming the ISTAF concept; (2) patients with ISTAF had a ≈3-fold risk of early death compared with patients with AF with no ischemic stroke after adjusting for confounders; and (3) the proportion of patients with ISTAF decreased, and their survival improved during the observation period despite their stable mean age and a slightly increased burden of stroke risk factors.

The temporal relationship between AF and ischemic stroke has most notably been suggested by a retrospective hospital-based study of 2605 patients,^6^ the Biomarkers for Cardiovascular Risk Assessment in Europe study combining a community cohort with 898 patients developing both AF and ischemic stroke during their follow-up,^7^ and 2 smaller earlier studies.^16,17^ The most recent of these studies suggested that more than one quarter of concurrent ischemic strokes and AF diagnoses occur within the 30 days preceding or following the ischemic stroke.^6,7^ Our study extends this knowledge by demonstrating in a large-scale population-based setting that first ischemic strokes in patients with AF cluster strongly within the 60-day time period. Interestingly, the proportion of ISTAF among all patients with AF decreased over time in our study. Explanations for this temporal change may be related not only to the aging of the population with more patients experiencing their first ischemic stroke before AF diagnosis but also to earlier detection of AF and improved anticoagulation practices that might have prevented ISTAFs. Our findings do not support this, but neither of them excludes systematic screening for AF. However, patients prone to AF should be encouraged to monitor their own heart rhythm and seek examinations readily if they notice a rhythm abnormality to prevent ischemic stroke as the first manifestation of their AF.

Our definition of the temporal connection between ischemic stroke and AF differs from most previous studies in which the starting point of the analysis has been hospitalization due to stroke. KAF was most often defined as AF known before stroke occurrence, but also diagnoses during hospital admission or hospital stay until 30 days have been regarded as KAF.^8–10,18^ Studies have defined AFDAS as AF diagnosed after these time windows with time windows defining new AFs ranging from a strict 7-day criteria^6^ to several months after stroke.^18^ Therefore, comparing our findings to studies using KAF and AFDAS definitions should be done cautiously. The ISTAF definition appears reasonable using comprehensive registry data as it accommodates the temporal distance between the 2 events and their entire distribution. Furthermore, after the extreme peak of ischemic strokes around the date of AF, frequencies of strokes appeared to plateau only >30 days prior and 30 days following AF. Patients with ISTAF also differed markedly in their clinical profile from both patients with past ischemic stroke and those with no ischemic stroke.

It is plausible to hypothesize that ISTAF events likely represent true cardiac embolism and not alternative stroke causes such as atherothrombosis or artery-to-artery embolism. It has also been proposed that a stroke can trigger AF by inducing an imbalance of sympathetic and parasympathetic activity or inflammation.^19^ Contrary to this assumption, a previous study showed that concomitant AF was rarely observed in patients with acute intracerebral hemorrhage compared with patients with acute ischemic stroke pointing out that AF clustering in ischemic strokes is most likely the true cause of stroke and not vice versa.^6^ Based on insights from pacemaker studies suggesting that increasing AF burden linearly increases the risk of ischemic stroke,^20^ it can be hypothesized that patients with ISTAF already have a profound burden of silent AF before they present with an acute ischemic stroke. Another hypothesis is that patients with ISTAF constitute a patient group with an extended thrombogenic atrial cardiomyopathy^21^ and ISTAF represents its first symptoms.

It is well known that among all subtypes of ischemic stroke, cardioembolic strokes—where AF is by far the leading high-risk embolic source—carry the highest risk of early mortality.^2^ In accordance with our findings, the Biomarkers for Cardiovascular Risk Assessment in Europe study found higher mortality early after AF diagnosis in patients with clustering ischemic stroke and AF, which then decreased after time elapsed from AF.^7^ However, studies dividing patients into KAF and AFDAS showed conflicting results, suggesting higher,^10^ lower,^6,8^ and similar^9^ mortalities in AFDAS patients compared with the KAF group. Study settings, patient populations, and the definitions used for the temporal relationship between AF and stroke may explain these discrepancies. In our study, patients with ISTAF had the worst survival, being an obvious finding due to the known high mortality carried by acute ischemic stroke. It has to be noted that our registry data could not be used for etiologic stroke subtyping, and it, thus, remains unknown whether ISTAFs predominantly represent cardioembolism or more diversely stroke etiologies. In fact, when systematically screened, new AF diagnoses are not uncommon among patients with acute strokes due to small-vessel occlusion and large-artery atherosclerosis.^22^ The effects carried by new-onset AF may also partly explain the higher mortality in patients with ISTAF, as increased mortality with new-onset AF has been shown also in other acute vascular conditions, such as myocardial infarction and pulmonary embolism.^23,24^

As a favorable trend, we showed improved survival rates over time, especially for patients with ISTAF, which supports our previous findings in the same cohort demonstrating a decrease in mortality in all patients with AF^25^ and large epidemiological studies showing improved stroke survival rates during the past decade.^26^ As risk factor burden and age of patients with ISTAF did not reduce over the years, potential explanations for the improved survival include better access to stroke unit care, higher utilization of recanalization therapies (intravenous thrombolysis and mechanical thrombectomy), and better control of cardiovascular risk factors. However, as shown in our analysis, anticoagulation initiation practices cannot explain the improved survival as there were no differences between the patient groups over the years, and only 5% of patients with ISTAF who died within 90 days had initiated any oral anticoagulation (0.4% with DOACs).

Our study has major strengths, the most obvious of which is the large nationwide population-based sample. We used multiple overlapping registries of high validity to define new-onset AF, ischemic stroke, and relevant comorbidities.^12^ Also, Finnish mortality data have been shown to be reliable.^23^ Limitations inherent to any registry study apply also to our study. We could not analyze AF subtype and specific stroke details, such as the severity of stroke symptoms and specific stroke cause, as such data are not collected directly in Finnish care registries. Furthermore, the dates for new-onset AF and ischemic stroke were based on the dates of the first registration of the diagnoses in different registries, so the dates we used do not necessarily refer to the exact onset dates of the events. It should also be mentioned that because we excluded a substantial proportion of the original cohort due to AF diagnosis or warfarin use between 2004 and 2006, this resulted in better accuracy in the selection and timing of new-onset AF cases. However, due to the improved diagnostics and treatment of AF, it may also have caused an underestimation of the number of patients with past ischemic stroke or ISTAF. Finally, our results apply to predominantly Whites and may not be directly generalizable to other populations.

In conclusion, a significant proportion of patients with new-onset AF experienced their first ischemic stroke at the same time as AF was diagnosed, forming the ISTAF presentation. The patients with ISTAF had higher mortality compared with patients with AF without past ischemic stroke or those with an ischemic stroke in their remote history. However, the reduction of patients with ISTAF and improvement in their survival rate over time appears a positive signal, for which underlying reasons warrant further research.

## ARTICLE INFORMATION

### Acknowledgments

The authors are indebted to Anu Eräkanto for her invaluable technical help in the article preparation. Dr Putaala: conceptualization, methodology, investigation, data curation, formal analysis, writing—original draft, and visualization and directly accessed and verified the underlying data reported in the article. Dr Teppo: conceptualization, methodology, data curation, writing—review and editing, and investigation. Dr Halminen: conceptualization, data curation, methodology, investigation, writing—review and editing, and project administration. Dr Haukka: conceptualization, methodology, data curation, writing—review and editing, and supervision. Dr Tiili: conceptualization, methodology, and writing—review and editing. Dr Jaakkola: conceptualization, methodology, and writing—review and editing. Dr Karlsson: conceptualization, methodology, and writing—review and editing. Dr Linna: conceptualization, methodology, and writing—review and editing. Dr Mustonen: conceptualization, writing—review and editing, and supervision. Dr Kinnunen: conceptualization, methodology, and writing—review and editing. Dr Aro: conceptualization, methodology, and writing—review and editing. Dr Kiviniemi: conceptualization, methodology, and writing—review and editing. Dr Hartikainen: conceptualization, methodology, writing—review and editing, and supervision. Dr Juhani Airaksinen: conceptualization, methodology, writing—review and editing, supervision, and project administration. Dr Lehto: conceptualization, methodology, writing—review and editing, supervision, project administration, and funding acquisition.

### Sources of Funding

This work was supported by the Aarne Koskelo Foundation, the Finnish Foundation for Cardiovascular Research, and the Helsinki and Uusimaa Hospital District Research Fund (TYH2019309).

### Disclosures

Dr Putaala: research grants: Helsinki and Uusimaa Hospital District, the Academy of Finland, the Finnish Foundation for Cardiovascular Research, the Sigrid Juselius Foundation, Bayer, and Amgen; speaker: Bayer, Boehringer Ingelheim, Bristol-Myers Squibb (BMS)-Pfizer, and Abbott; advisory board: Novo Nordisk and Herantis Pharma; visiting editor: Terve Media; and stock ownership: Vital Signum. Dr Teppo: research grants: the Finnish Foundation for Cardiovascular Research and the Aarne and Aili Turunen Foundation. Dr Haukka: consultant: Research Janssen R&D; speaker: Bayer Finland; and research grants: the Jane and Aatos Erkko Foundation, Avohoidon Tutkimussäätiö, and the European Commission (Brussels; 282526). Dr Karlsson: research grants: AstraZeneca Heart Scholarship granted by the Finnish Cardiac Society. Dr Linna: speaker: BMS-Pfizer, Bayer, and Boehringer Ingelheim. Dr Mustonen: consultant: Roche, BMS-Pfizer alliance, Novartis Finland, Boehringer Ingelheim, and MSD Finland. Dr Kiviniemi: research grants: EU/Horizon-EIC-Pathfinder, EU/Horizon2020/Business Finland-Moore4Medical, the Finnish Medical Foundation, the Finnish Foundation for Cardiovascular Research, and Hospital District of Southwest Finland. Dr Hartikainen: research grants: the Finnish Foundation for Cardiovascular Research, EU Horizon 2020, and EU FP7; advisory board member: BMS-Pfizer alliance, Novo Nordisk, and Amgen; and speaker: Cardiome and Bayer. Dr Juhani Airaksinen: research grants: the Finnish Foundation for Cardiovascular Research; speaker: Bayer, Pfizer, and Boehringer Ingelheim; and advisory board: Bayer, Pfizer, and AstraZeneca. Dr Lehto: research grants: the Aarne Koskelo Foundation, the Finnish Foundation for Cardiovascular Research, Helsinki and Uusimaa Hospital District, and Boehringer Ingelheim; consultant: BMS-Pfizer, Bayer, Boehringer Ingelheim, and MSD Finland; and speaker: BMS-Pfizer, Bayer, Boehringer Ingelheim, MSD Finland, Terve Media, and Orion Pharma. The other authors report no conflicts.

### Supplemental Material

Tables S1–S4

Figures S1–S8

## Supplementary Material


