# Supplementary material for: Ischemic Stroke Temporally Associated With New-Onset Atrial Fibrillation: A Population-Based Registry-Linkage Study
Source: Stroke. 2023 Dec 8;55(1):122–30. doi: 10.1161/STROKEAHA.123.044448 (PMC10734779; doi:10.1161/STROKEAHA.123.044448)

SUPPLEMENTAL MATERIAL

Ischemic stroke temporally associated with incident atrial fibrillation: A population-based registry-linkage study

Authors:

Jukka Putaala, MD, PhD[1]; Konsta Teppo, MD[2]; Olli Halminen, MSc, PhD[3]; Jari Haukka, PhD[4]; Paula Tiili, MD[1]; Jussi Jaakkola, MD, PhD[2]; Elin Karlsson, MD[5]; Miika Linna, PhD[3]; Pirjo Mustonen, MD, PhD[2]; Janne Kinnunen, MD[1]; Tuomas Kiviniemi, MD, PhD[2]; Aapo Aro, MD, PhD[6]; Juha Hartikainen, MD, PhD[7]; K.E. Juhani Airaksinen, MD, PhD[2]; Mika Lehto, MD, PhD[8]; on behalf of the FinACAF study group.

Affiliations:

[1]Department of Neurology, Helsinki University Hospital and University of Helsinki, Finland.
[2]Heart Center, Turku University Hospital and University of Turku, Finland.
[3]Department of Industrial Engineering and Management, Aalto University, Espoo, Finland.
[4]Department of Public Health, University of Helsinki, Finland.
[5]University of Helsinki, Finland.
[6]Heart and Lung Center, Helsinki University Hospital and University of Helsinki, Finland.
[7]Heart Center, Kuopio University Hospital and University of Eastern Finland, Kuopio, Finland.
[8]Department of Internal Medicine, Jorvi Hospital and Helsinki University Hospital, Espoo, Finland and University of Helsinki, Helsinki, Finland.

Supplemental Table S1. Definitions of comorbidities used in the present analysis.

	**ICD-10**	**ICPC-2**	**Reimbursement code**	**ATC code**
Hypertension	I10-I15	K85, K86, K87	205	C03A, C03B, C03DB, C03EA, C07A, C08CA, C08D, C09
Dyslipidemia	E78	T93	206	C10
Heart failure	I50, I11.0, I13.0, I13.2	K77	201	
Diabetes	E10-E14	T89, T90	103, 215	A10
Vascular disease	I20-I25, I65-I66, I67.2, I70	K74, K75, K76, K91, K92	206	
Prior bleeding	D50.0, D62, D68.3, I60-I62, I69.0-I69.2, I85.0, I86.4, J94.2, K22.1, K22.3, K22.6, K25.0, K25.2, K25.4, K25.6, K26.0, K26.2, K26.4, K26.6, K27.0, K27.2, K27.4, K27.6, K28.0, K28.2, K28.4, K28.6,			

	K29.0, K62.5, K63.1, K63.3, K92.0-K92.2, N02, R04, R31, R58, S06.2-S06.6, S06.8			
Alcohol use disorder	F10			
Chronic kidney disease	N18, Z49			
Liver dysfunction (liver cirrhosis or failure)	K70.2-K70.4, K71.7, K71.8, K72, K74			
Dementia	F00-F03, G30			
Psychiatric disorder	F32, F33, F34.1, F40-F42, F43.1, F31, F20	P76, P74, P73, P72		

ATC, anatomic therapeutic chemical; ICD-10, International Classification of Diseases, Tenth Revision; ICPC-2, International Classification of Primary Care, Second Edition.

Supplemental Table S2. Distribution of modified CHA_2DS_2-VASc score in patients with incident atrial fibrillation (AF) stratified by occurrence and timing of ischemic stroke in relation to AF.

	**No ischemic stroke (n=204 774)**	**Past ischemic stroke (n=12 209)**	**Ischemic stroke temporally with AF (n=12 582)**
**Modified CHA_2DS_2-VASc points**			
0	7.0	0	2.6
1	12.8	0	7.9
2	17.5	0.7	15.3
3	20.7	3.9	23.3
4	22.2	9.6	29.0
5	13.6	19.2	15.3
6	5.2	30.1	5.7
7	1.0	23.2	0.9
8	0	10.7	0
9	0	2.5	0
**Modified CHA_2DS_2-VASc groups**			
0-1	19.8	0	10.5
2	17.5	0.7	15.3
3	20.7	3.9	23.3
4	22.2	9.6	29.0
5-9	19.8	85.7	21.9

Modified CHA_2DS_2-VASc, congestive heart failure, hypertension, age ≥75 years, diabetes, *history of stroke >30 days prior to incident AF*, vascular disease, age 65–74 years, sex category (female).

Supplemental Table S3. Logistic regression analysis on variables associated with incident ischemic stroke temporally with incident atrial fibrillation (AF) among patients without past ischemic stroke. All the displayed variables were forced into the model, which was further adjusted for cohort entry year.

**Covariate**	**Adjusted odds ratio (95% confidence interval)**
Age group	
<65 years	Reference
65-74 years	1.939 (1.824-2.061)
≥75 years	2.767 (2.605-2.938)
Female sex	1.013 (0.974-1.052)
Level of education	
Category 1	1.283 (1.216-1.353)
Category 2	1.162 (1.096-1.233)
Category 3	Reference
Vascular disease (yes vs. no)	0.880 (0.842-0.920)
Diabetes (yes vs. no)	0.987 (0.943-1.033)
Dyslipidemia (yes vs. no)	1.069 (1.026-1.113)
Heart failure (yes vs. no)	0.672 (0.637-0.709)
Hypertension (yes vs. no)	1.061 (1.014-1.111)
Prior bleeding (yes vs. no)	1.085 (1.024-1.150)
Liver dysfunction (yes vs. no)	0.786 (0.578-1.070)
Chronic kidney disease (yes vs. no)	0.911 (0.825-1.007)
Alcohol use disorder (yes vs. no)	1.193 (1.061-1.340)
Cancer (yes vs. no)	0.833 (0.796-0.872)
Dementia (yes vs. no)	0.989 (0.914-1.070)
Psychiatric disorder (yes vs. no)	0.952 (0.892-1.016)

Supplemental Table S4. Cox regression analysis on factors associated with 90-day all-cause mortality among patients with incident ischemic stroke temporally associated with atrial fibrillation. All displayed variables were forced into the model, which was further adjusted for cohort entry year.

**Covariate**	**Adjusted hazard ratio (95% confidence interval)**
Age group	
<65 years	Reference
65-74 years	1.770 (1.359-2.306)
≥75 years	4.069 (3.180-5.207)
Female sex	1.053 (0.955-1.161)
Level of education	
Category 1	1.231 (1.060-1.430)
Category 2	0.999 (0.836-1.195)
Category 3	Reference
Vascular disease (yes vs. no)	1.289 (1.162-1.431)
Diabetes (yes vs. no)	1.164 (1.043-1.299)
Dyslipidemia (yes vs. no)	0.162 (0.552-0.679)
Heart failure (yes vs. no)	2.160 (1.945-2.399)
Hypertension (yes vs. no)	1.045 (0.931-1.173)
Prior bleeding (yes vs. no)	1.218 (1.070-1.386)
Liver dysfunction (yes vs. no)	4.191 (2.582-6.803)
Chronic kidney disease (yes vs. no)	1.312 (1.076-1.601)
Alcohol use disorder (yes vs. no)	0.809 (0.567-1.155)
Cancer (yes vs. no)	1.175 (1.058-1.304)
Dementia (yes vs. no)	1.858 (1.618-2.133)
Psychiatric disorder (yes vs. no)	1.078 (0.921-1.261)

Supplemental Figure S1. Flow chart of patient selection. ICD-10, International Classification of Diseases, Tenth Revision; OAC, oral anticoagulation; FinACAF, Finnish AntiCoagulation in Atrial Fibrillation; AF, atrial fibrillation.

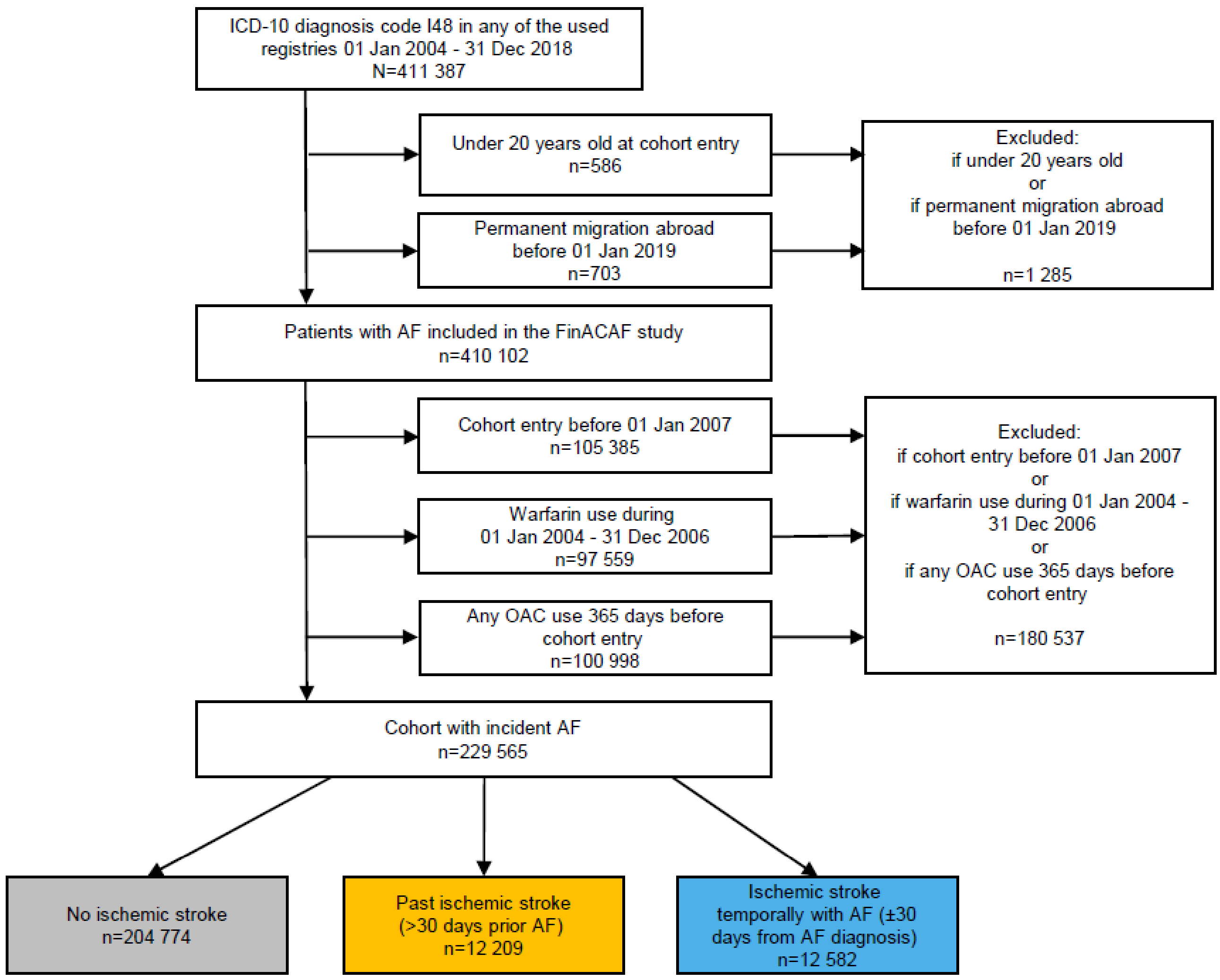

Supplemental Figure S2. Frequency of incident ischemic strokes according to temporal distance (months) from incident atrial fibrillation (AF) among all patients in the study (n=229 565).

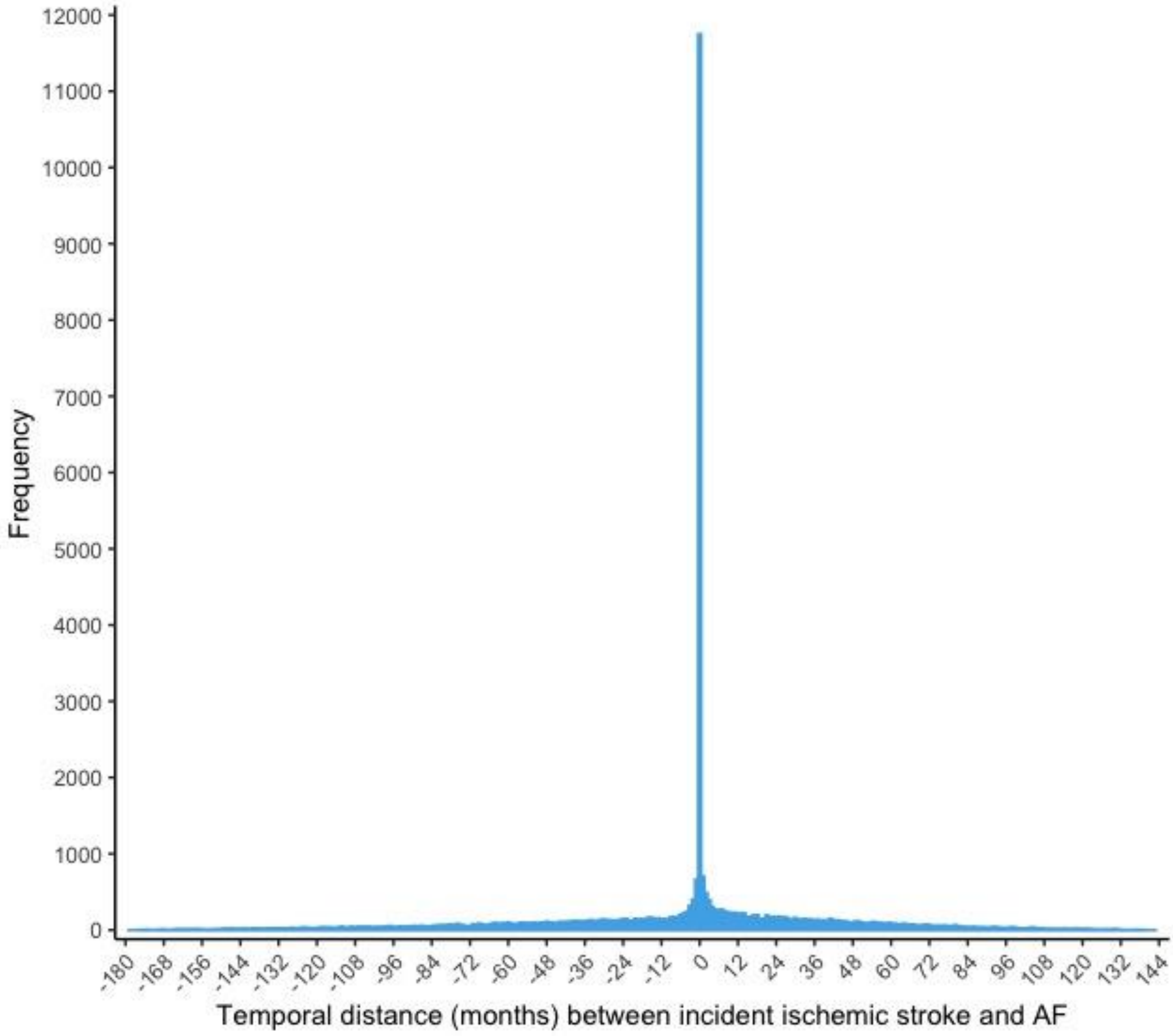

Supplemental Figure S3. Frequency of patients with incident atrial fibrillation (AF) in subgroups stratified by temporal relationship between ischemic stroke and AF over time (p-value <0.001 for trends).

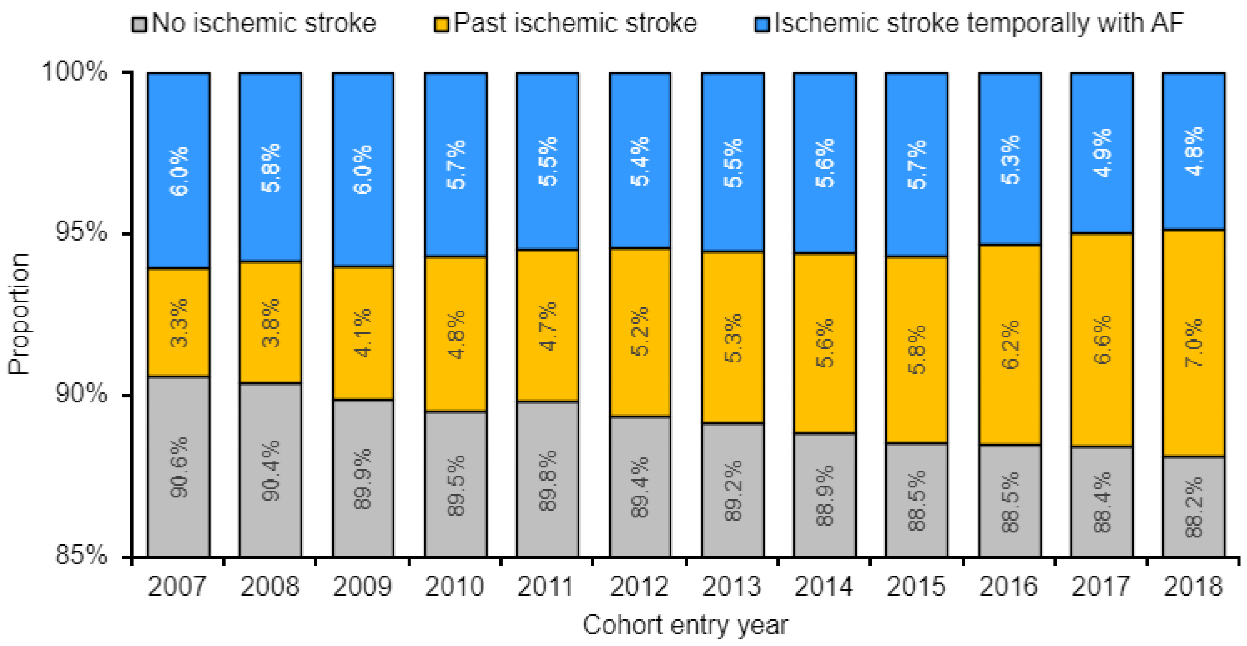

Supplemental Figure S4. Annual mean age during 2007-2018 stratified by phenotype based on temporal relationship between ischemic stroke and atrial fibrillation (AF). Shaded areas mark 95% confidence intervals.

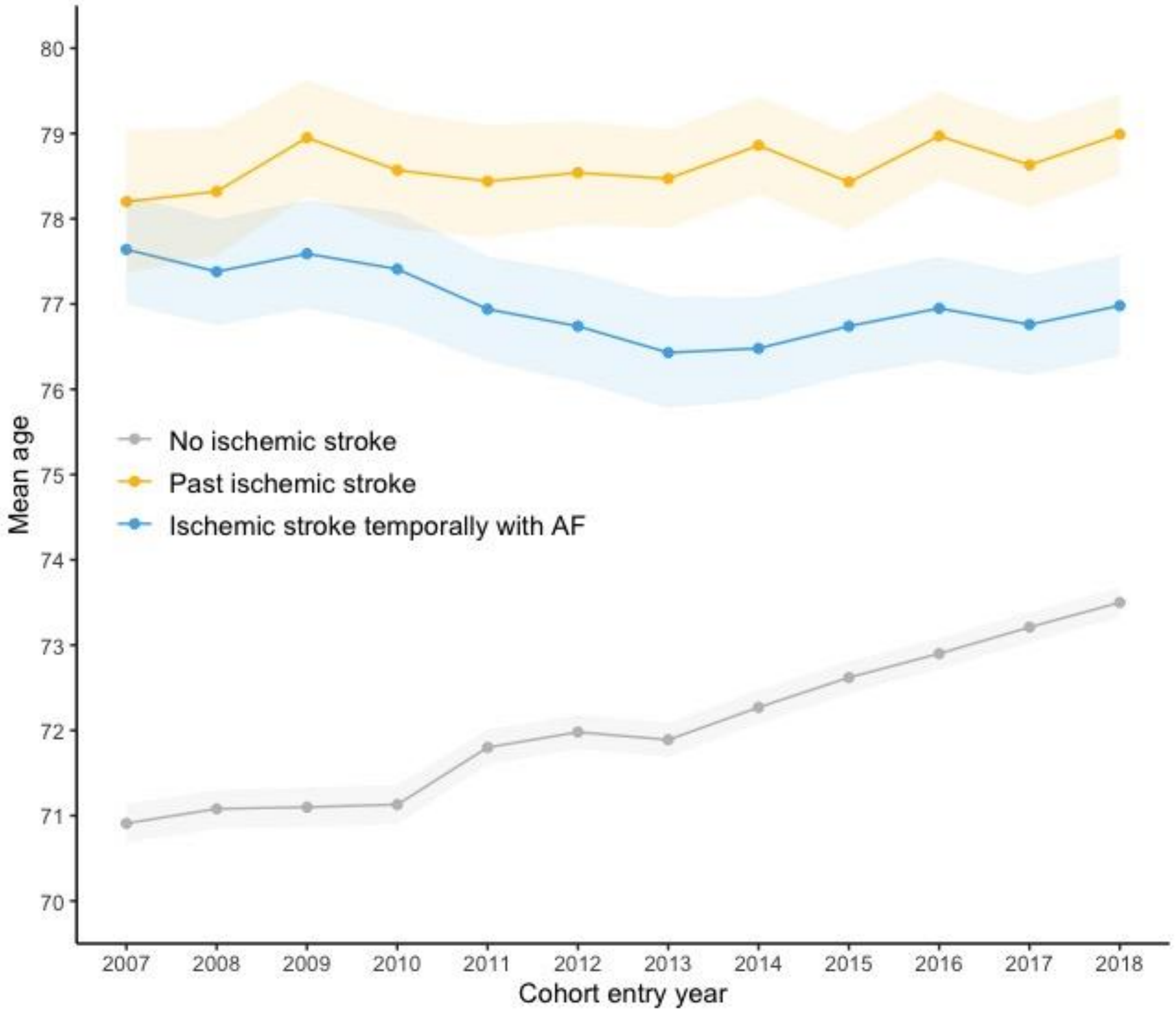

Supplemental Figure S5. Frequency of patients with incident atrial fibrillation (AF) in modified CHA_2DS_2-VASc subgroups stratified by phenotype based on temporal relationship between ischemic stroke and AF.

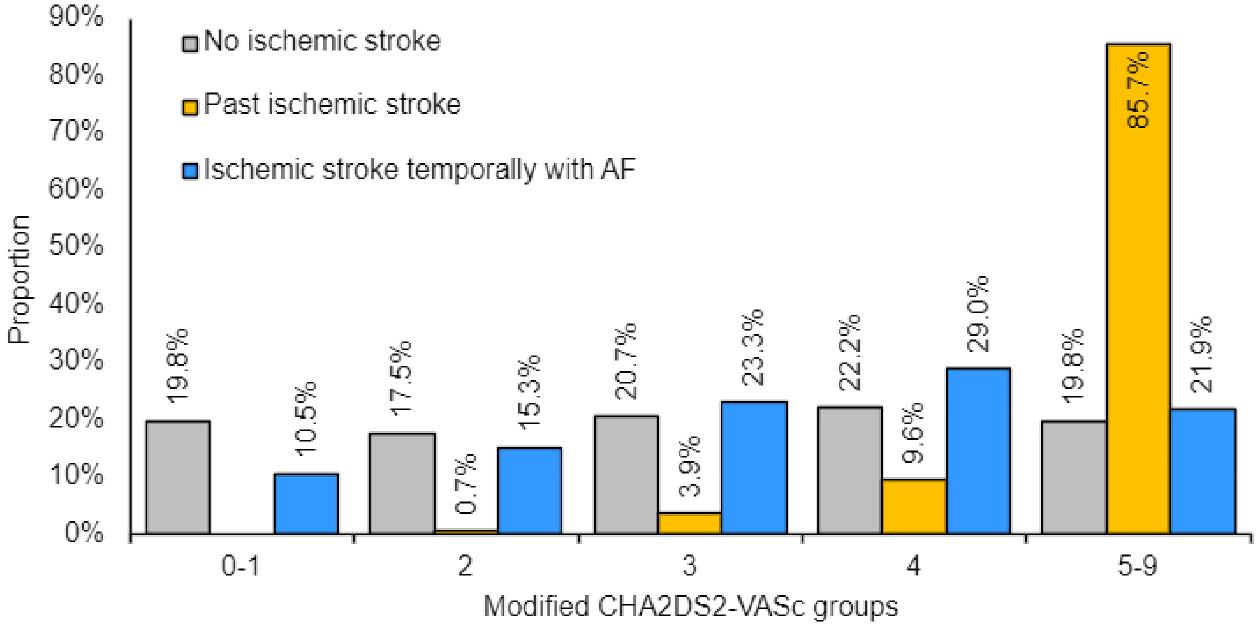

Supplemental Figure S6. Annual mean CHA_2DS_2-VASc score during 2007-2018 stratified by phenotype based on temporal relationship between ischemic stroke and atrial fibrillation (AF). Mann-Kendall p-value for trends were <0.001, 0.03, 0.001 for mean CHA_2DS_2-VASc score in patients with no ischemic stroke, past ischemic stroke, and ischemic stroke temporally with AF, respectively.

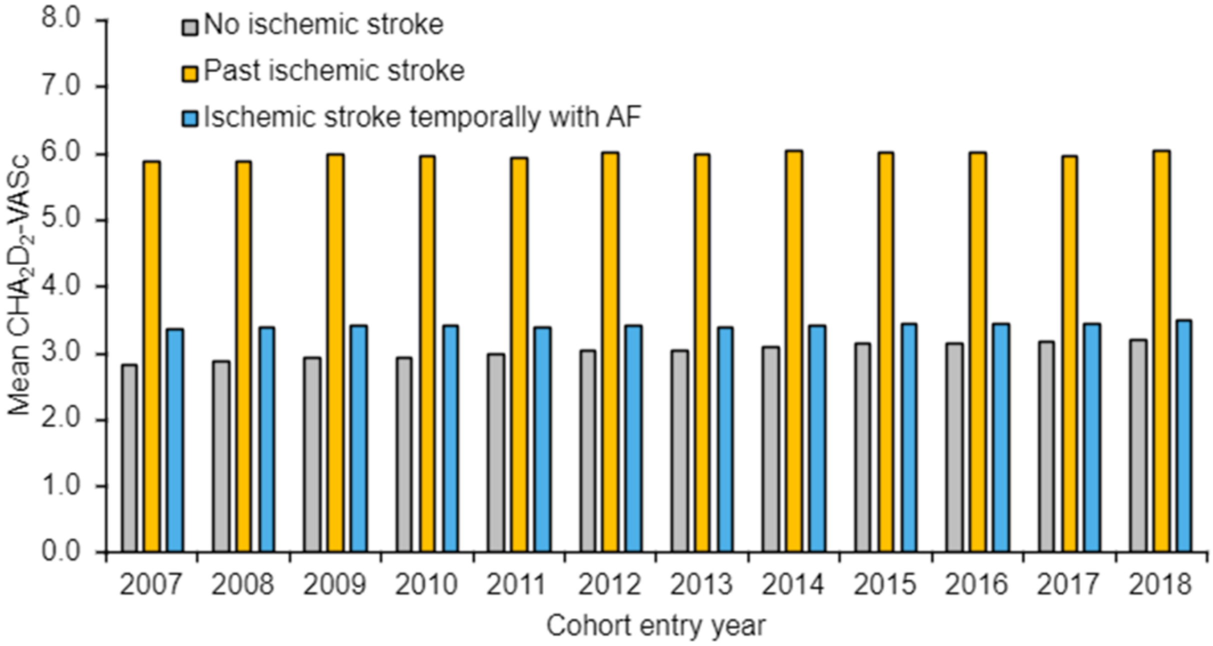

Supplemental Figure S7. Annual proportions of study patients initiating warfarin and direct oral anticoagulants (DOACs) during 2007-2018 stratified by temporal relationship between ischemic stroke and atrial fibrillation (AF).

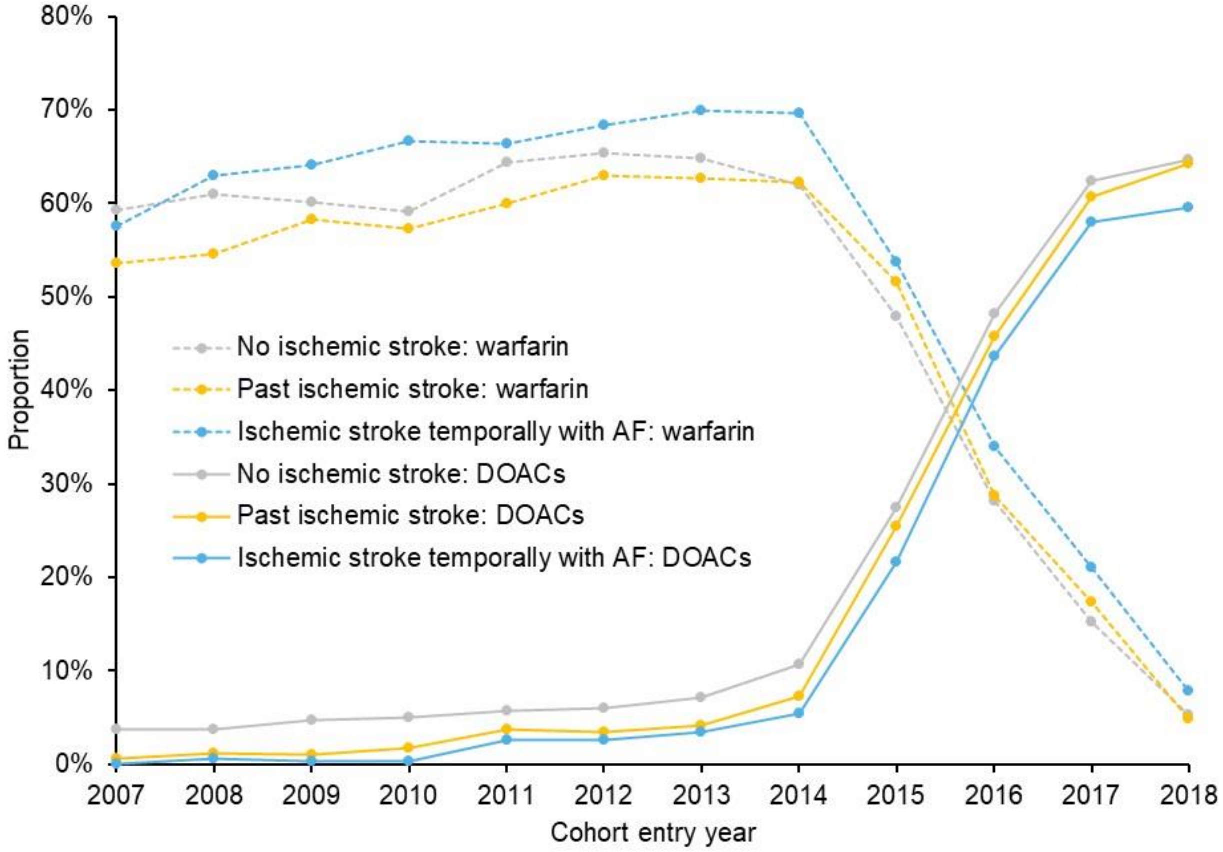

Supplemental Figure S8. Kaplan Meier curve on 90-day survival in patients with incident ischemic stroke temporally associated with atrial fibrillation according to modified CHA_2DS_2-VASc score (log rank $p<0.001$). Shaded areas denote 95% confidence intervals.

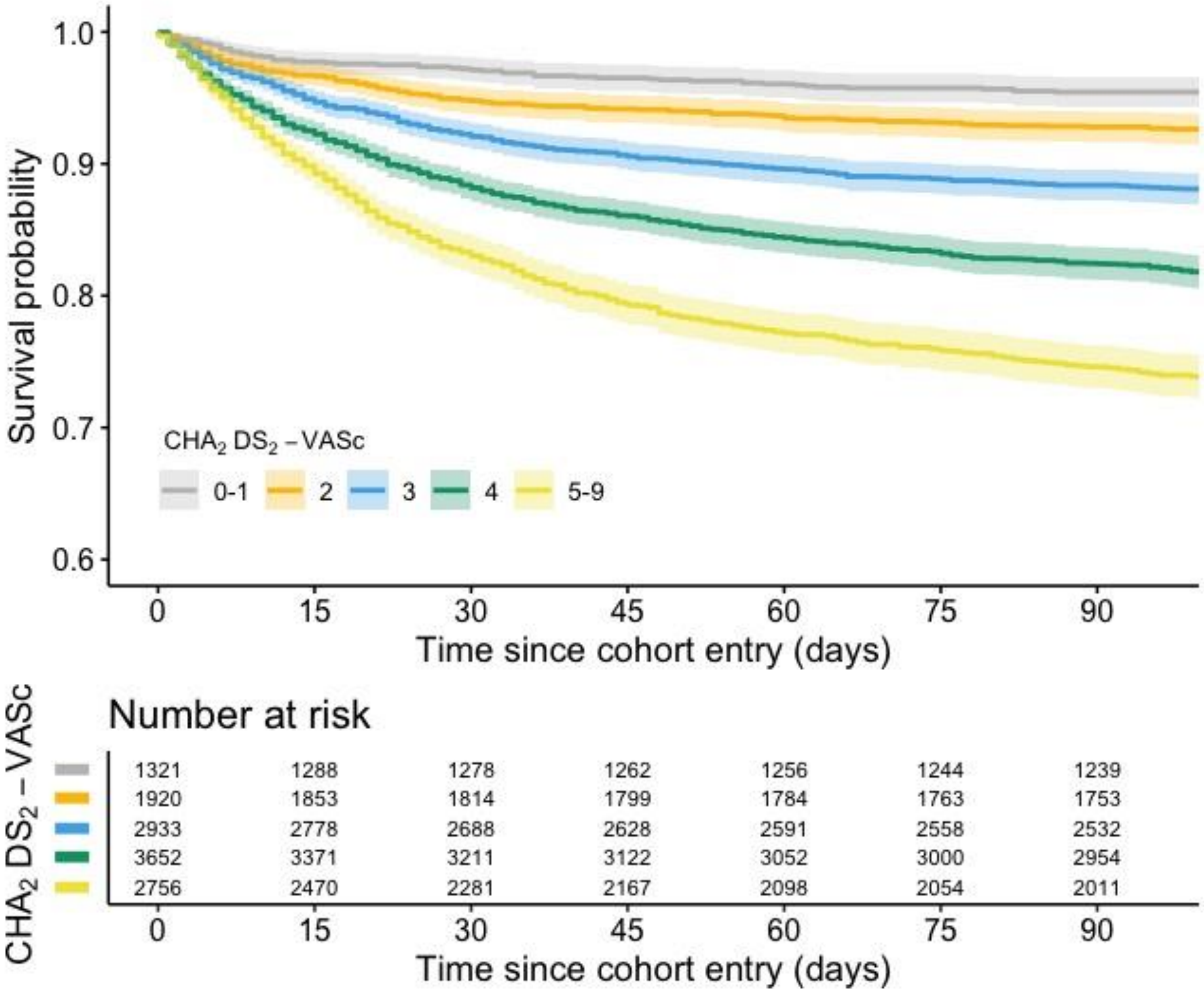

Supplement: Supplementary file 1 [file str-55-122-s001.pdf]
